# Evaluation of Metabolic Dysfunction-Associated Fatty Liver Disease-Related Pathogenic Mechanisms in Human Steatotic Liver Cell-Based Model: Beneficial Effects of *Prunus domestica* L. subsp. *syriaca* Extract

**DOI:** 10.3390/nu17071249

**Published:** 2025-04-03

**Authors:** Laura Comi, Claudia Giglione, Fationa Tolaj Klinaku, Lorenzo Da Dalt, Hammad Ullah, Maria Daglia, Paolo Magni

**Affiliations:** 1 Department of Pharmacological and Biomolecular Sciences, Università degli Studi di Milano, 20133 Milan, Italy; laura.comi1@unimi.it (L.C.); claudia.giglione@unimi.it (C.G.); fationa.tolaj@unimi.it (F.T.K.); lorenzo.dadalt@unimi.it (L.D.D.); 2 School of Pharmacy, University of Management and Technology, Lahore 54000, Pakistan; hammadrph@gmail.com; 3 Department of Pharmacy, University of Naples Federico II, 80168 Naples, Italy; maria.daglia@unina.it; 4 International Research Center for Food Nutrition and Safety, Jiangsu University, Zhenjiang 212013, China; 5 IRCCS MultiMedica, 20099 Sesto San Giovanni, Milan, Italy

**Keywords:** metabolic dysfunction-associated fatty liver disease (MASLD), HepG2 hepatocytes, oleic acid, glucose uptake, oxidative stress, lipid accumulation, *Prunus domestica* L. subsp. *syriaca*, natural extract, agro-food waste

## Abstract

**Background/Objectives**: Disrupted glucose uptake, oxidative stress, and increased de novo lipogenesis are some of the key features of metabolic dysfunction-associated fatty liver disease (MASLD). The modulation of these pathogenic mechanisms using extracts from natural and sustainable sources is a promising strategy to mitigate disease progression. This study aimed to evaluate the effects of *Prunus domestica* L. subsp. syriaca extract on these processes, taking advantage of a cell-based model of steatotic hepatocytes (HepG2-OA) that recapitulates some key pathophysiological features of MASLD. **Methods**: The HepG2-OA cell model was generated by treating cells for 7 days with 100 μM oleic acid (OA). The effect of different concentrations (0.01, 0.1, 0.5, and 1 mg/mL) of *P. domestica* extract was assessed through MTT assay (cell viability), flow cytometry (glucose uptake and reactive oxygen species, ROS, production), spectrophotometry (lipid accumulation), and qRT-PCR (expression of selected genes). **Results**: *P. domestica* extract exhibited no cytotoxicity at any tested concentration after 24 and 48 h in the HepG2-OA cells. The extract increased glucose uptake in a dose-dependent fashion after both 6 and 24 h. Additionally, the extract reduced lipid accumulation and downregulated the expression of key lipogenic genes (DGAT1 and FASN). Furthermore, in the HepG2-OA cells, *P. domestica* extract reduced ROS production and downregulated the expression of oxidative stress-related genes (SOD and CAT). **Conclusions**: *P. domestica* extract positively modulated some key molecular mechanisms associated with glucose metabolism, lipogenesis, and oxidative stress, supporting its potential as a nutraceutical candidate for MASLD management.

## 1. Introduction

Metabolic dysfunction-associated fatty liver disease (MASLD) has emerged as the most prevalent chronic liver condition worldwide [1], affecting approximately one in four individuals [2]. Closely linked to obesity and type 2 diabetes mellitus (T2DM), MASLD represents a critical component of metabolic syndrome and is projected to rise in prevalence alongside these conditions [3]. While often asymptomatic in its early stages, MASLD can progress to metabolic dysfunction-associated steatohepatitis (MASH), a more severe form characterized by inflammation, hepatocellular damage, and fibrosis, increasing the risk of cirrhosis and hepatocellular carcinoma (HCC) [4].

In this context, cell-based in vitro models are crucial for evaluating the effect of potential treatments for MASLD on specific pathogenetic mechanisms. Interestingly, when exposed to the fatty acid oleic acid (OA), human HepG2 cells, a widely used cell model able to mimic some hepatocyte features [5], develop intracellular lipid accumulation and cytotoxic responses, closely recapitulating some key features of MASLD [6]. Specifically, this cell-based model, defined here as HepG2-OA, recapitulates the relevant disease’s molecular hallmarks, including fat accumulation [7], driven by de novo lipogenesis, insulin resistance, oxidative stress, and inflammation [8].

Current MASLD management relies on lifestyle modifications, particularly the adoption of a Mediterranean diet [9] and regular physical activity, which improve insulin sensitivity and reduce liver fat [10]. Given the absence of approved pharmacological treatments [11], nutraceuticals, derived from food sources, are being explored for their potential hepatoprotective effects [12]. Polyphenols [13], omega-3 fatty acids, probiotics, prebiotics [14], and silymarin [15] have demonstrated antioxidant and anti-inflammatory properties on HepG2 steatotic cells, proving effective for MASLD management [16].

*Prunus domestica* L. subsp. *syriaca* (*P. domestica*; Mirabelle plum) extract is a promising nutraceutical candidate in this field due to its rich composition of hydroxycinnamic acids, flavanols, and glycoside flavonols, which contribute to its antioxidant and anti-inflammatory activities [17]. Various *Prunus* species have been shown to positively regulate body weight, glucose and lipid homeostasis, insulin resistance, prothrombin state, and chronic inflammation, as well as improve hypertension-related signaling pathways [18]. Furthermore, since the plum production chain features a relevant amount of agro-food waste, utilizing *Prunus* extracts aligns with sustainable agro-food waste valorization, adding economic and environmental benefits [19].

This study aimed to test the effect of *P. domestica* extract on the modulation of lipid accumulation, the reduction in glucose uptake, and the increase in oxidative stress, taking advantage of the HepG2-OA cell model.

## 2. Materials and Methods

### 2.1. Chemicals

Dulbecco’s Phosphate-Buffered Saline (DPBS), Fetal Bovine Serum (FBS), L-Glutamine, non-essential amino acids, dimethyl sulfoxide (DMSO), and isopropanol (99.5%) were obtained from Gibco ThermoFisher (Waltham, MA, USA). Oleic Acid-Water Soluble, hydrogen peroxide (H_2_O_2_), 6-Hydroxy-2,5,7,8-tetramethylchroman-2-carboxylic acid (Trolox), Lipid (Oil Red O) Staining kit, and dextran-coated charcoal (DCC) were obtained from Sigma-Aldrich (Milan, Italy). A total of 100 mM sodium pyruvate, 1X Trypsin–EDTA in PBS, Eagle’s Minimum Essential Medium (MEM), 100X penicillin–streptomycin solution, and Moon kit’s Universal One-Step RT-qPCR Kit (E3005) were obtained from EuroClone (Milan, Italy). 3-(4,5-dimethylthiazol-2-yl)–2,5-diphenyl tetrazolium bromide (MTT), CellROX^TM^ Green Reagent for oxidative stress detection (C10444), and 6-(*N*-(7-Nitrobenz-2-oxa-1,3-diazol-4-yl)amino)-6-Deoxyglucose (6-NBDG) were obtained from Invitrogen (Waltham, MA, USA). Direct-zol RNA Miniprep Kit and TRI Reagent^®^ were obtained from Zymo Research (Irvine, CA, USA).

### 2.2. Cell Cultures and Differentiation

The human HepG2 hepatocellular carcinoma cell line (ATCC^®^ HTB-92™) was obtained from the American Type Culture Collection (ATCC^®^, Manassas, VA, USA) and cultured according to the recommended protocols. The cells were grown and maintained in MEM supplemented with 2.0 mM L-Glutamine, 0.1 mM non-essential amino acids, 1.0 mM sodium pyruvate, 10,000 U/mL penicillin, 10 mg/mL streptomycin, and 10% FBS.

To obtain a steatotic in vitro model mimicking some MASLD features, HepG2 cells were treated with 100 μM OA for 7 days, following the guidelines in the literature [20]. Specifically, OA was added 24 h after cell seeding in multiwell plates and was replenished with every medium change (occurring every 2 or 3 days). After 7 days of OA treatment, the cells (designated as HepG2-OA) were considered fully differentiated into the steatotic phenotype, making them suitable for subsequent experiments. During the experimental exposure to *P. domestica* extract, H_2_O_2_, or Trolox, OA was not added, as the cells had already undergone full steatotic differentiation. The cells were cultured in 100 mm Petri dishes or in 96-, 24-, or 6-multiwell plates at 37 °C in a humidified atmosphere with 5% CO_2_.

### 2.3. Preparation of Prunus domestica L. subsp. syriaca Extract

The *P. domestica* extract was prepared following the method described by Ullah et al. [17]. Briefly, fruit samples were sourced from a local farm in the Campania Region, Italy. The fruits were thoroughly washed with water to remove any dirt or residues. The skins were peeled off, and the pulp was cut into small pieces with a ceramic knife. To prevent oxidation during preparation, the samples were cut in an ice bath. The prepared pulp was freeze-dried and ground into a fine powder using a mortar and pestle. Aliquots of 2 g of the powdered pulp were added to 40 mL of 50% hydroethanolic solution acidified with a 0.1% HCl solution (adjusted to pH 2.0). The mixture was subjected to magnetic stirring for 3 h at room temperature, followed by centrifugation at 6000 rpm for 10 min. The precipitate was separated from the supernatant, and the extraction process was repeated three times. The collected supernatants were filtered through Whatman cellulose filter paper. The filtrate was then concentrated using a rotary evaporator and was subsequently freeze-dried. Given the high glucose and sucrose content identified via nuclear magnetic resonance (NMR) spectroscopy [17], the dry extract underwent a chemical precipitation of its sugars. This was achieved by a treatment with absolute ethanol, followed by ultra-freezing temperatures. The organic solvent was then removed under reduced pressure using a rotary evaporator. The resulting sugar-free dry extract of the *P. domestica* fruit pulp was stored at −20 °C for subsequent biological assays.

For the cell treatment experiments, the *P. domestica* extract was reconstituted in DMSO to a stock concentration of 200 mg/mL. Serial dilutions were prepared to achieve concentrations of 100, 20, and 2 mg/mL in DMSO. These diluted solutions were then added to a DCC-treated culture medium, resulting in final working concentrations of 1, 0.5, 0.1, and 0.01 mg/mL, which were used across all experiments.

### 2.4. 3-(4,5-Dimethylthiazol-2-yl)-2,5-diphenyltetrazolium Bromide MTT Cell Vitality Assay

Cell viability was assessed using the MTT proliferation assay, following a previously established protocol [21]. This colorimetric assay relies on the reduction of yellow tetrazolium salt (MTT) to insoluble purple formazan via metabolically active cells. HepG2-OA cells were treated with *P. domestica* at specified concentrations. After 24 and 48 h, MTT was added directly to each well. The resulting formazan crystals were then dissolved by adding DMSO. Absorbance was measured at 570 nm using an EnSpire Multimode Plate Reader spectrophotometer (PerkinElmer Italia, Milano, Italy) with a dynamic range from 0.000 to 0.200 O.D. Cell viability was calculated using the following formula:

$$\mathrm{Cell}\;\mathrm{Viability}\;\%=\frac{\left(\mathrm{Average}\;\mathrm{ABS}\;\mathrm{Samples}\;-\;\mathrm{Average}\;\mathrm{ABS}\;\mathrm{Blank}\right)}{\left(\mathrm{Average}\;\mathrm{ABS}\;\mathrm{Controls}\;-\;\mathrm{Average}\;\mathrm{ABS}\;\mathrm{Blank}\right)}\;\times \;100$$

### 2.5. Lipid (Oil Red O) Quantification

The assay was performed using Oil Red O staining, a dye that selectively binds to neutral lipids, allowing for their visualization and quantification. Oil Red O staining was used for the morphological staining of the control HepG2-OA cells and cells treated with *P. domestica* at the concentration of 1 mg/mL (grown on cover glass and stained as described below), as well as for lipid quantification in the HepG2-OA cells. To this aim, the cells were seeded in a 12-well plate for morphological staining and in a 96-well plate for lipid quantification and treated with *P. domestica* extract at the specified concentrations. After 24 h, the cells were fixed with 10% formalin, followed by staining with an Oil Red O working solution. The excess stain was removed with 60% isopropanol, and hematoxylin was used for counterstaining. The images were obtained using a phase contrast optical microscope at 40× magnification. Absorbance was measured at 492 nm using an EnSpire PerkinElmer Multimode Plate Reader spectrophotometer, with a dynamic range from 0.000 to 0.200 O.D.

### 2.6. Glucose Uptake NovoCyte3000 Flow Cytometer Analysis

Glucose uptake in the HepG2-OA cells was assessed using 6-NBDG, a fluorescent glucose analog that allows for the quantification of cellular glucose uptake via flow cytometry. A total of 72 and 96 h after seeding, the cells were treated with *P. domestica* extract at the various concentrations. Following 6 or 24 h of treatment, the cells were incubated with 20 µM 6-NBDG for 30 min. After incubation, the cells were collected and analyzed using a NovoCyte 3000 flow cytometer (Agilent Technologies Italia, Milano, Italy) [22].

### 2.7. Reactive Oxygen Species (ROS) NovoCyte3000 Flow Cytometer Analysis

ROS production in the HepG2-OA cells was evaluated using the fluorogenic probe CellROX^TM^ Green Reagent (Thermo Fisher Scientific, Milano, Italy), which detects oxidative stress by binding to DNA in the presence of reactive oxygen species (ROS), resulting in a strong green fluorescence signal. The HepG2-OA cells were treated with *P. domestica* extract at various concentrations, as well as with Trolox (500 μM) as a positive control. After 24 h of treatment, the cells were incubated with 5 μM CellROX^TM^ Green Reagent, followed by 500 μM H_2_O_2_ for 30 min, to induce oxidative stress. The cells were then collected for analysis using a NovoCyte3000 flow cytometer.

### 2.8. Reverse Transcription–Quantitative Polymerase Chain Reaction (RT-qPCR)

To evaluate the expression of the genes involved in lipid metabolism (DGAT1, diacylglycerol acyltransferase-1, and FASN, fatty acid synthase) and oxidative stress (SOD, superoxide dismutase, and CAT, catalase), RT-qPCR was performed. HepG2-OA cells were treated with different concentrations of *P. domestica* extract for 6 or 24 h. After treatment, the cells were collected for RNA extraction and purification using a Direct-zol RNA kit (Minipreps; Zymo Research, Irvine, CA, USA). RNA purity and concentration were assessed using a NanoDrop spectrophotometer (Thermo Fisher Scientific, Milano, Italy). The mRNA levels of the target genes listed in Table 1 were measured using a Luna Universal One-Step RT-qPCR kit (E3005; EuroClone, Milano, Italy) according to the provided protocol. ACTB, β-actin, was used as the housekeeping gene for normalization.

### 2.9. Statistical Analysis

The results from three independent experiments carried out in triplicate were expressed as the mean percentage (%) ±SD (standard deviation) compared to a control. The graphs and data were analyzed using GraphPad Prism version 9.0.2. The statistical analysis was conducted with One-Way ANOVA or Two-Way ANOVA multiple comparisons, followed by a Dunnett test, considered statistically significant at *p* < 0.05.

## 3. Results

### 3.1. HepG2-OA Cell Viability Was Not Affected by P. domestica Extract Treatment (24 and 48 h)

The MTT cell viability assay was conducted to evaluate the potential cytotoxic effects of different concentrations of *P. domestica* treatments on HepG2-OA cells. The concentrations tested were 0.01, 0.1, 0.5, and 1 mg/mL. The main objective was to determine the appropriate concentrations for further experimental assays while maintaining cell viability. Following previous validation studies [21], concentrations that caused a reduction in cell viability below 80% compared to control cells (CTRs), which were untreated and assumed to have 100% viability, were considered toxic. The MTT assay measures cell metabolic activity as an indicator of viability, where live cells convert the MTT reagent into formazan crystals, resulting in a color change that is spectrophotometrically measurable.

**Figure 1:**
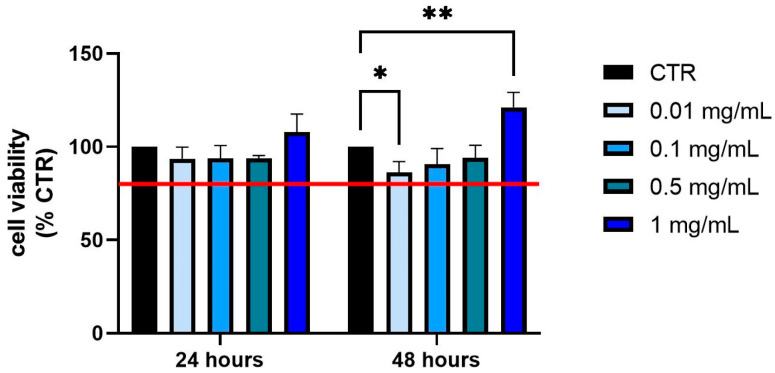
Effect of treatment with *P. domestica* at concentrations of 0.01, 0.1, 0.5, and 1 mg/mL for 24 and 48 h on HepG2-OA cell viability. The red line at 80% indicates the threshold above which cells can be considered viable. The data are expressed as a percentage, representing the mean ± SD (standard deviation), and normalized to the control (CTR = 100%). The graph was obtained from the average of three separate experiments, each performed in quadruplicate. * *p* < 0.05; ** *p* < 0.01 (Two-Way ANOVA multiple comparison, Dunnett test).

The results showed that none of the *P. domestica* treatments at the tested concentrations caused a significant decrease in cell viability below the 80% threshold, even after 24 and 48 h of exposure in HepG2-OA (Figure 1). Specifically, after 48 h of treatment at the 0.01 mg/mL *P. domestica* concentration, a significant reduction in cell viability was observed (*p* < 0.05), although viability remained above the 80% threshold. In contrast, the 1 mg/mL treatment at the same time point resulted in a significant increase in cell viability (*p* < 0.01), suggesting a positive effect at this concentration. These findings indicate that the tested concentrations of *P. domestica* are non-toxic and suitable for further experiments. This provides a solid foundation for the exploration of the potential therapeutic effects of *P. domestica* in future assays.

### 3.2. Lipid Content and Expression of Genes Involved in Lipid Metabolism in HepG2-OA Were Reduced by P. domestica Extract

**Figure 2:**
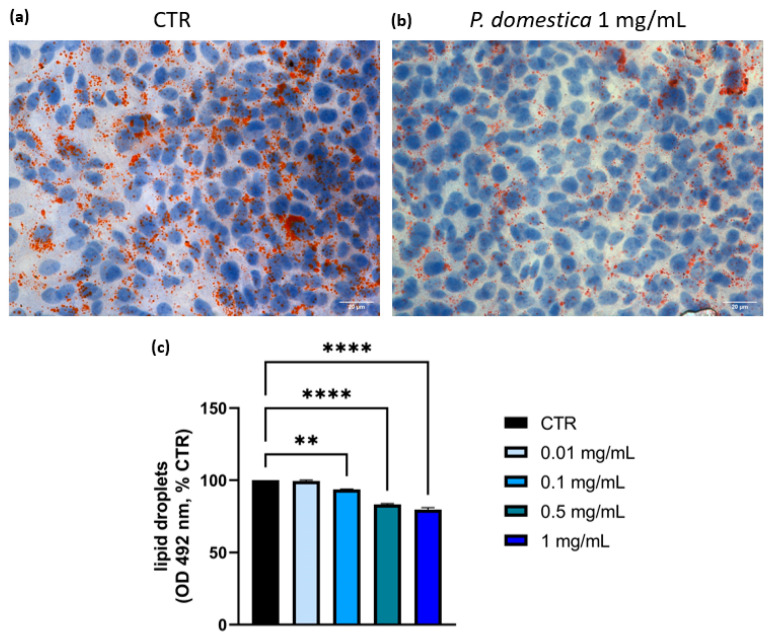
Oil Red O staining in HepG2-OA control cells and in HepG2-OA cells treated with 1 mg/mL of *P. domestica* extract for 24 h, and effect of 24 h treatment with *P. domestica* extract on lipid accumulation in HepG2-OA cells. (**a**) Oil Red O staining of control (CTR) HepG2-OA, showing a marked lipid accumulation after OA treatment (40× magnification). Nuclei were stained in blue with hematoxylin. (**b**) Oil Red O staining of HepG2-OA cells treated with 1 mg/mL of *P. domestica* extract (40× magnification). Nuclei were stained in blue with hematoxylin. (**c**) Effect of treatment with *P. domestica* on lipid accumulation in HepG2-OA cell line. Quantification of lipids was performed using “EnSpire PerkinElmer Multimode Plate Reader” spectrophotometer. Data are presented as mean ± SD (standard deviation) and normalized to control (CTR = 100%, corresponding to 199.1 ng/µL). Graph represents average of three separate experiments, each conducted in triplicate. ** *p* < 0.01; **** *p* < 0.0001 (One-Way ANOVA multiple comparison, Dunnett test).

Lipid accumulation, a hallmark of MASLD, was assessed in HepG2-OA cells using Oil Red O staining, comparing control cells with those treated with *P. domestica* extract at a concentration of 1 mg/mL. As shown in Figure 2a, HepG2-OA showed a high basal high intracellular lipid content, as evidenced by the intense red staining of lipid droplets, corresponding to a lipid concentration of 199.1 ng/µL. Figure 2b shows that treatment with 1 mg/mL of *P. domestica* extract significantly reduced lipid droplet accumulation compared to the control.

Following a 24 h treatment with *P. domestica* extract, a dose-dependent reduction in lipid content was observed in the HepG2-OA cells (Figure 2c). Specifically, lipid accumulation decreased by 6.4% at 0.1 mg/mL (*p* < 0.01), reaching a maximum reduction of 20.3% at 1 mg/mL (*p* < 0.0001), indicating a partial recovery from OA-induced steatosis.

These findings suggest that *P. domestica* extract partially counteracts OA-induced lipid accumulation, supporting its potential role in mitigating this process.

The potential modulation of gene expression following exposure to *P. domestica* extract at different concentrations for 6 and 24 h was assessed using RT-qPCR in HepG2-OA cells. The analysis specifically focused on the expression of the DGAT1 gene, a key enzyme involved in triglyceride synthesis, and the FASN gene, which plays a central role in lipogenesis. The gene expression levels were normalized to the housekeeping gene ACTB.

**Figure 3:**
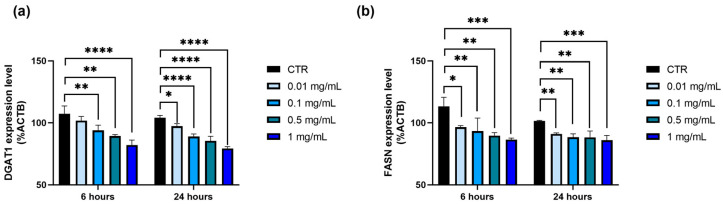
Lipid metabolism-related gene expression in HepG2-OA cells treated for 6 or 24 h with *P. domestica* extract. (**a**) Expression levels of diacylglycerol O-acyltransferase 1 (DGAT1) after 6 and 24 h of treatment with *P. domestica* in HepG2-OA cells. (**b**) Expression levels of fatty acid synthase (FASN) after 6 and 24 h of treatment with *P. domestica* in HepG2-OA cells. Experiments were conducted using RT-qPCR, and results are expressed as percentages, representing mean of three separate experiments, each performed in triplicate. Values are normalized against expression levels of housekeeping gene β-actin (ACTB) (ACTB = 100%). * *p* < 0.05; ** *p* < 0.01; *** *p* < 0.001; **** *p* < 0.0001 (Two-Way ANOVA multiple comparison, Dunnett test).

Following treatment with *P. domestica* extract, the DGAT1 expression in HepG2-OA cells decreased in a dose-dependent manner, with a significant reduction observed after both 6 and 24 h (Figure 3a). After 6 h, DGAT1 gene expression was significantly reduced by 12.6% (0.1 mg/mL; *p* < 0.01), reaching a maximum decrease of 23.6% (1 mg/mL; *p* < 0.0001), compared to the control. After 24 h, the reduction ranged from 6.5% (0.01 mg/mL; *p* < 0.05) to 23.7% (1 mg/mL; *p* < 0.0001) compared to the control.

Similarly, the FASN gene expression in HepG2-OA cells (Figure 3b) was significantly downregulated in a dose-dependent manner at both 6 and 24 h of exposure to *P. domestica* extract. After 6 h, the reduction ranged from 14.9% (0.01 mg/mL; *p* < 0.05) to 23.7% (1 mg/mL; *p* < 0.001), while after 24 h, it varied between 10.4% (*p* < 0.01) and 15.3% (*p* < 0.001) at the same concentrations.

### 3.3. HepG2-OA Glucose Uptake Increased in a Dose-Dependent Fashion After 6 and 24 h P. domestica Extract Treatments

The impact of *P. domestica* treatment on the glucose uptake in HepG2-OA cells was assessed. A quantitative analysis was performed using a flow cytometer, which measures the fluorescent signal emitted by the glucose labeled with the fluorophore 6-NBDG that was absorbed by the cells. The cells were exposed to different concentrations of *P. domestica* extract for 6 and 24 h.

**Figure 4:**
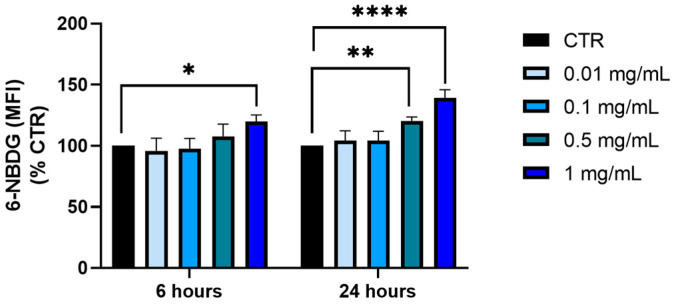
Glucose uptake in HepG2-OA cells following treatment with *P. domestica* extract at concentrations of 0.01, 0.1, 0.5, and 1 mg/mL for 6 and 24 h. The evaluations were conducted using the NovoCyte3000 flow cytometer. The graph represents the mean of three separate experiments, each performed in triplicate. The data are expressed as mean fluorescent intensity (MFI) percentages. Each bar indicates the mean ± SD (standard deviation), with the control set at 100%. * *p* < 0.05, ** *p* < 0.01; **** *p* < 0.0001 (Two-Way ANOVA multiple comparison, Dunnett test).

Upon treatment with the *P. domestica* extract, these cells showed a dose-dependent increase in glucose uptake (Figure 4). Compared to the control, glucose uptake increased by 19.7% after 6 h of treatment at the highest concentration tested (1 mg/mL; *p* < 0.05) and by 20.4% (*p* < 0.01) and 39.1% (*p* < 0.0001) at concentrations of 0.5 mg/mL and 1 mg/mL, respectively, after 24 h.

### 3.4. Reactive Oxygen Species (ROS) Production and the Expression of Genes Involved in Oxidative Stress Were Reduced by P. domestica Extract in HepG2-OA Cells

This study also explored the potential impact of *P. domestica* extract on ROS production and the expression of genes related to oxidative stress in HepG2-OA cells due to their relevance in MASLD. ROS production was quantitatively measured using flow cytometry after treatment with *P. domestica* extract at the concentrations of 0.01, 0.1, 0.5, and 1 mg/mL.

**Figure 5:**
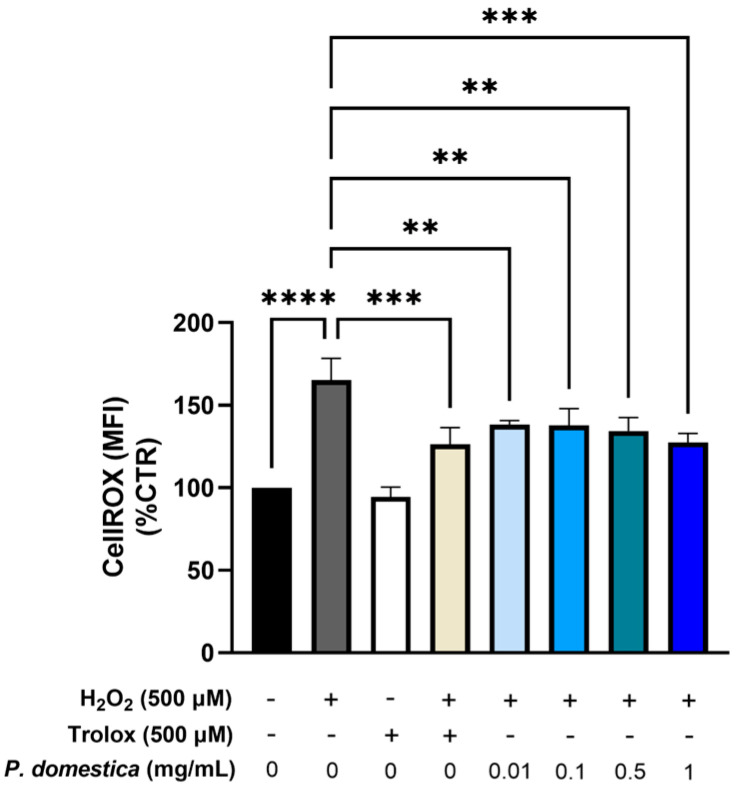
Intracellular formation of reactive oxygen species (ROS) in HepG2-OA cells treated with *P. domestica* extract for 24 h at different concentrations. The quantification of ROS was performed using a NovoCyte3000 flow cytometer. The data are presented as mean fluorescent intensity (MFI) and are normalized to the control (CTR = 100%). This Figure shows the average of three separate experiments, each conducted in triplicate. ** *p* < 0.01; *** *p* < 0.001; **** *p* < 0.0001 (One-Way ANOVA multiple comparison, Dunnett test).

In the HepG2-OA cells, a 500 µM H_2_O_2_ treatment was used as a pro-oxidant control, resulting in a 65.2% (*p* < 0.0001) increase in ROS production compared to the control cells. Trolox, a potent antioxidant, was able to only partially restore the control ROS levels upon H_2_O_2_ challenge (−23.7%, *p* < 0.001). Interestingly, the treatments with *P. domestica* at various concentrations resulted in a significant reduction in ROS production, with a decrease ranging from 16.4% at 0.01 mg/mL (*p* < 0.01) to 22.9% at 1 mg/mL (*p* < 0.001) (Figure 5), suggesting that the extract was unable to fully quench the H_2_O_2_-induced ROS production in this cell model.

To further investigate the effects of *P. domestica* extract, RT-qPCR was used to assess the expression of genes related to oxidative stress, specifically SOD and CAT, in response to *P. domestica* treatment. The expression levels of these genes were normalized to ACTB as a housekeeping gene.

**Figure 6:**
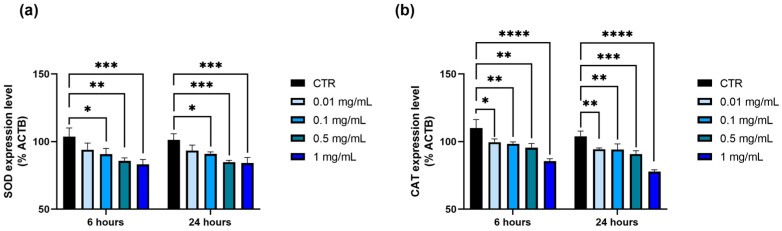
Expression levels of genes involved in oxidative stress in HepG2-OA cells treated with *P. domestica* extract at different concentrations and at different time points. (**a**) Expression levels of superoxide dismutase (SOD) after 6 and 24 h of treatment with *P. domestica* in HepG2-OA cells. (**b**) Expression levels of catalase (CAT) after 6 and 24 h of treatment with *P. domestica* in HepG2-OA cells. Experiments were conducted using RT-qPCR, and results are expressed as percentages representing mean of three separate experiments, each performed in triplicate. Values are normalized against expression levels of housekeeping gene β-actin (ACTB) (ACTB = 100%). * *p* < 0.05; ** *p* < 0.01; *** *p* < 0.001; **** *p* < 0.0001 (Two-Way ANOVA multiple comparison, Dunnett test).

In the HepG2-OA cells, *P. domestica* treatment resulted in a significant, dose-dependent decrease in SOD gene expression at both 6 and 24 h. After 6 h, reductions ranged from 12.6% at 0.1 mg/mL (*p* < 0.05) to 19.8% at 1 mg/mL (*p* < 0.001), and after 24 h, reductions of 10.2% (*p* < 0.05) and 16.9% (*p* < 0.001) were observed at the same concentrations, respectively (Figure 6a).

For CAT gene expression, a significant dose-dependent decrease was observed in the HepG2-OA cells, with the most notable reduction at 1 mg/mL after 6 (−22.2%; *p* < 0.0001) and 24 h (−25%; *p* < 0.0001) (Figure 6b).

## 4. Discussion

MASLD is an escalating global health concern arising from the convergence of multiple pathogenetic mechanisms. Unhealthy dietary habits, a high fructose intake, and a sedentary lifestyle represent important background conditions of MASLD and may exacerbate disease progression [23]. Additionally, visceral obesity [24], T2DM [1], dyslipidemia [25], and arterial hypertension [26] are well-established risk factors promoting MASLD onset and progression, with genetic predispositions, such as PNPLA3 and TM6SF2 [27] polymorphisms, further increasing susceptibility. Notably, MASLD patients face an elevated risk of cardiovascular diseases, including heart disease and stroke, due to overlapping metabolic dysfunctions [28]. Given the chronic nature of the disease and its limited pharmacological options, nutraceutical interventions hold promise for both prevention and management. Exploring the effects of novel, sustainable plant extracts on selected mechanisms relevant to MASLD pathogenesis by using experimental cell-based models of human origin can provide valuable insights before clinical assessment and application. Importantly, the use of a sustainable extract also aligns with the principles of the UN 2030 Agenda for Sustainable Development and the One Health concept.

In this study, we took advantage of a cell-based model of steatotic human hepatocytes (HepG2-OA) to explore the effects of a sustainable extract derived from *Prunus domestica* L. subsp. *syriaca*. Our findings underscore the pathophysiological relevance of targeting lipid dysregulation, glucose imbalance, and oxidative stress in MASLD, highlighting the potential of *P. domestica* extract as an effective nutraceutical approach. Our results demonstrated that treatment with *P. domestica* extract significantly reduced lipid content and downregulated the expression of genes involved in lipid biosynthesis. Additionally, the extract enhanced glucose uptake in a dose-dependent manner in HepG2-OA cells. Furthermore, treatment with *P. domestica* extract effectively reduced ROS production and modulated the expression of genes related to oxidative stress in these cells, reinforcing its potential as a nutraceutical agent in MASLD management.

Importantly, *P. domestica* extract did not affect cell viability, confirming that the selected concentrations were suitable for the experiments. This approach ensured experimental consistency and enhanced the relevance of our findings for future applications.

Lipid accumulation in hepatocytes is a key feature of MASLD, driven by disrupted lipid metabolism and metabolic stress [8]. Notably, *P. domestica* extract reduced both gene expression levels and lipid accumulation in a dose-dependent manner in HepG2-OA cells, highlighting its potential in mitigating this hallmark of MASLD. DGAT1 is a key enzyme in lipid homeostasis, and in MASLD, its overactivity drives excessive lipid production, contributing to hepatic steatosis and to metabolic disturbances like lipotoxicity, oxidative stress, and inflammation [29]. The extract’s ability to reduce DGAT1 expression levels is consistent with findings on other polyphenolic compounds [30], such as green tea catechins, which inhibit hepatic lipogenesis by directly targeting lipid synthesis pathways [31]. Additionally, the anthocyanins in *P. domestica*, known for their lipid-lowering properties, likely contribute to DGAT1 inhibition, further reducing lipid buildup in hepatocytes [32]. FASN gene expression, another key enzyme in lipogenesis, was also significantly suppressed by the *P. domestica* extract, aligning with the actions of other polyphenolic compounds such as resveratrol [33] and quercetin [34], which inhibit fatty acid synthesis by downregulating FASN expression and modulating transcription factors like sterol regulatory element-binding protein 1c, SREBP-1c, a critical regulator of lipid metabolism [35]. These findings highlight the potential of *P. domestica* extract, particularly its anthocyanin and catechin content, in addressing lipid dysregulation. By downregulating the genes involved in lipid metabolism and reducing lipid accumulation, the extract targets the mechanisms responsible for excessive hepatic lipid buildup. A limitation of this study is the lack of data on the potential contribution of genes related to lipolysis in the observed lipid content reduction, such as PPARα/γ, CPT1, and SREBP-1c, which play essential roles in lipid oxidation and synthesis. Nevertheless, given the complexity of MASLD pathophysiology, these findings suggest that *P. domestica* extract may be useful for prevention or as part of a combined therapeutic approach. Its lipid-lowering effects, especially when used alongside agents targeting inflammation, fibrosis, or insulin resistance, could provide a more comprehensive strategy for managing the disease.

Impaired glucose metabolism is another hallmark of MASLD [36]. Notably, *P. domestica* extract significantly enhanced glucose uptake in HepG2-OA cells in a dose-dependent manner, with a maximal effect of +39.1% at the highest concentration tested (1 mg/mL). This effect is likely attributable to the extract’s flavonoid content, as flavonoids are known to enhance glucose uptake and improve insulin sensitivity [37]. Compounds such as quercetin and anthocyanins, present in *P. domestica*, are particularly effective in improving insulin signaling pathways [38], thereby mitigating insulin resistance, a major driver of MASLD progression. These findings are consistent with research on related compounds like berberine [39] and curcumin [40], which similarly enhance hepatic glucose uptake and insulin sensitivity by modulating pathways such as AMP-activated protein kinase (AMPK) [41] and insulin receptor substrate 1 (IRS-1) [42]. This effect is particularly relevant to MASLD, where the interplay between glucose and lipid dysregulation exacerbates disease severity. Given the liver’s central role in maintaining glucose balance, the ability of *P. domestica* extract to enhance glucose uptake in HepG2-OA cells suggests its potential to address key metabolic derangements associated with MASLD.

The antioxidant properties of *P. domestica* represent a significant aspect of its protective potential, especially considering the critical role that oxidative stress plays in MASLD progression [43]. The ROS produced in hepatocytes can lead to cellular damage, inflammation, and fibrosis, advancing the condition from simple steatosis to MASH [44]. MASH is more severe as persistent inflammation results in the deposition of extracellular matrix proteins, leading to fibrosis and liver dysfunction [45]. In turn, advanced fibrosis disrupts liver architecture and function, potentially progressing to cirrhosis, liver failure, and an increased risk of liver cancer [46]. In our experimental model, the ROS production increase induced by H_2_O_2_ treatment in HepG2-OA cells reached a very high extent (+65.5%), possibly recapitulating the elevated oxidative stress characteristic of steatotic liver conditions. The *P. domestica* extract partially but significantly reduced ROS production in a dose-dependent manner, with up to a 22.9% ROS decrease in HepG2-OA cells. The observed antioxidant effects of *P. domestica* are likely attributed to its high anthocyanin content, known to directly neutralize ROS [47] and influence antioxidant enzyme activity in hepatocyte models [48,49]. These effects align with findings from studies on anthocyanin-rich fruits like blueberries and black currants [50]. Additionally, the flavonoids and phenolic acids in *P. domestica* may enhance the body’s antioxidant defenses [51] by boosting the activity of endogenous antioxidant enzymes. Our study also demonstrated that *P. domestica* extract modulated the expression of oxidative stress-related genes, specifically downregulating SOD and CAT gene expression in association with reduced oxidative stress. SOD, a key antioxidant enzyme, dismutates superoxide radicals into hydrogen peroxide and oxygen, preventing oxidative damage. This reduction in SOD expression likely reflects a decreased oxidative burden, as lower ROS levels reduce the need for heightened SOD activity [52]. This regulatory effect is consistent with other polyphenolic compounds, such as those from green tea [53] and curcumin [54], which similarly downregulate oxidative stress-related genes as ROS levels decline, thus protecting liver cells from oxidative damage and inflammation. Likewise, CAT, another crucial antioxidant enzyme, decomposes hydrogen peroxide into water and oxygen, preventing oxidative damage. The observed reduction in CAT expression aligns with decreased ROS levels and suggests that lower oxidative stress reduces the need for enhanced CAT activity [55]. This regulatory effect mirrors findings from other studies on polyphenolic compounds, which similarly downregulated oxidative stress-related enzymes following ROS reduction. For instance, salicylic acid treatment was shown to enhance CAT activity and gene expression levels, thereby enhancing antioxidant capacity [56]. Similarly, tart cherry pit extracts modulated CAT gene expression, contributing to their antioxidant effects [57]. By reducing ROS levels and altering the expression of oxidative stress-related genes, *P. domestica* extract may help preserve cellular function and integrity in the context of MASLD. This dual action positions *P. domestica* as a promising treatment agent for the mitigation of oxidative damage and the improvement in metabolic function in in vitro liver disease models at least.

Beyond its biological efficacy, *P. domestica* extract exemplifies the innovative reuse of agro-food waste, aligning with the One Health approach, which underscores the interconnectedness of human, animal, and environmental health [58]. By valorizing agricultural and food processing by-products, this extract promotes a circular economy while minimizing environmental impacts. This sustainability-focused strategy directly supports the United Nations’ 2030 Agenda for Sustainable Development, advancing multiple Sustainable Development Goals (SDGs) [59]. It enhances food security by reducing waste and optimizing resource efficiency, contributing to SDG 2 (Zero Hunger) [60]. The health-promoting properties of *P. domestica* extract, including its antioxidant and metabolic benefits, align with SDG 3 (Good Health and Well-being) by aiding in the prevention and management of non-communicable diseases [61]. Additionally, by minimizing food waste and promoting sustainable agricultural practices, *P. domestica* extract advances SDG 12 (Responsible Consumption and Production) while also mitigating environmental impacts such as greenhouse gas emissions, thus supporting SDG 13 (Climate Action) [62].

## 5. Conclusions

In conclusion, this study highlighted the potential of *P. domestica* extract in positively modulating pathogenic pathways that are hallmarks of MASLD, including lipid metabolism, glucose uptake, and oxidative stress. *P. domestica*’s rich polyphenolic content underscores its promise as an accessible and effective nutraceutical for MASLD management, particularly for patients with limited treatment options.

One limitation of this study is the use of just an in vitro model, which, while providing a cost-effective and reproducible platform for the assessment of metabolic dysfunction related to MASLD, does not fully capture the complexity of liver pathology. Additionally, the direct bioavailability of the *P. domestica* extract to hepatic cells remains uncertain, as the digestion and absorption processes were not considered in this study. Future studies should incorporate bioaccessible fractions obtained through in vitro digestion or relevant metabolites to better understand the extract’s physiological relevance. Furthermore, these findings underscore the need for further investigation into additional mechanisms, such as insulin sensitivity, glucose transporter (GLUT) trafficking, and other oxidative stress regulators like Nuclear factor erythroid 2-related factor 2, Nrf2. Moreover, given that MASLD is a chronic condition requiring prolonged intervention, future studies should assess the extract’s long-term effects beyond the 48 h time frame used in this study to better evaluate its safety and efficacy.

With the rising prevalence of MASLD and the demand for sustainable treatment solutions, *P. domestica* extract represents a promising addition to nutraceutical-based strategies for liver disease prevention and management, potentially slowing disease progression and improving patient outcomes.

## Figures and Tables

**Table 1:** Forward and reverse sequences of the primers used.

Gene	Forward Sequence (5′→3′)	Reverse Sequence (3′→5′)
ACTB	CACCATTGGCAATGAGCGGTTC	AGGTCTTTGCGGATGTCCACGT
FASN	TATGCTTCTTCGTGCAGCAGTT	GCTGCCACACGCTCCTCTAG
DGAT1	AACTGGTGTGTGGTGATGCT	CCTTCAGGAACAGAGAAACC
SOD	GGTGTGGGGAAGCATTAAAGG	CAAGTCTCCAACATGCCTCTC
CAT	TTTAACGCCATTGCCACAGG	TGAGGCCAAACCTTGGTGA

## Data Availability

The data presented in this study are available on request from the corresponding author.
